# The role of the purposeful shared decision making model in vascularized composite allotransplantation

**DOI:** 10.3389/frtra.2024.1421154

**Published:** 2024-07-09

**Authors:** Ian G. Hargraves, Kasey R. Boehmer, Hatem Amer, Cassie C. Kennedy, Joan M. Griffin, Dawn M. Finnie, Victor M. Montori, Fantley Clay Smither, Samir Mardini, Steven Moran, Sheila Jowsey-Gregoire

**Affiliations:** ^1^ Knowledge and Encounter Research (KER) Unit, Mayo Clinic, Rochester, MN, United States; ^2^ Division of Nephrology and Hypertension, Mayo Clinic, Rochester, MN, United States; ^3^ Division of Health Care Delivery Research, Mayo Clinic, Rochester, MN, United States; ^4^ Robert D. and Patricia E. Kern Center for the Science of Health Care Delivery, Mayo Clinic, Rochester, MN, United States; ^5^ Division of Pulmonary and Critical Care Medicine, Mayo Clinic, Rochester, MN, United States; ^6^ William J. von Liebig Center for Transplantation and Clinical Regeneration, Mayo Clinic, Rochester, MN, United States; ^7^ Department of Physical Medicine and Rehabilitation, Mayo Clinic, Rochester, MN, United States; ^8^ Division of Plastic and Reconstructive Surgery, Department of Surgery, Mayo Clinic, Rochester, MN, United States; ^9^ Department of Psychiatry and Psychology, Mayo Clinic, Jacksonville, FL, United States

**Keywords:** shared decision making (SDM), Purposeful SDM, vascularized composite allotransplantation, VCA, patient clinician communication, patient centered care, model

## Abstract

For some patients who have lost the lower part of an arm, hand transplant offers the possibility of receiving a new limb with varying degrees of sensation and function. This procedure, Vascularized Composite Allotransplantation (VCA), is demanding for patients and their care community and comes with significant risks. As a high-stakes decision, patients interested in VCA are subject to extensive clinical evaluation and eligibility decision making. Patients and their care community must also decide if hand transplant (versus other approaches including rehabilitative therapies with or without prosthesis) is right for them. This decision making is often confusing and practically and emotionally fraught. It is complicated in four ways: by the numerous beneficial and harmful potential effects of hand transplant or other options, the number of people affected by VCA and the diverse or conflicting positions that they may hold, the practical demands and limitations of the patient's life situation, and the existential significance of limb loss and transplant for the patient's being. Patients need support in working through these treatment determining issues. Evaluation does not provide this support. Shared decision making (SDM) is a method of care that helps patients think, talk, and feel their way through to the right course of action for them. However, traditional models of SDM that focus on weighing possible beneficial and harmful effects of treatments are ill-equipped to tackle the heterogeneous issues of VCA. A recent model, Purposeful SDM extends the range of troubling issues that SDM can help support beyond opposing effects, to include conflicting positions, life situations, and existential being. In this paper we explore the pertinence of these issues in VCA, methods of SDM that each require of clinicians, the benefits of supporting patients with the breadth of issues in their unique problematic situations, implications for outcomes and practice, and extend the theory of the Purposeful SDM model itself based on the issues present in hand transplant decision making.

## 1 Introduction

Patients who live with bi-lateral upper extremity limb loss may experience many significant and life diminishing medical, psychological, and sociological challenges. These may include loss of function and occupation (1), depression (2), dependency on others (3), and social isolation (4), to name only a few. Unlike solid organ transplantation which offers a life saving solution of end stage diseases, upper extremity transplantation provides a life enhancing strategy for those with limb loss. The implications of transplantation are considerable as these patients will be exposed to the risks of life long immunosuppressive medication for which they must also adjust their life to accommodate rigid daily administration schedules, and, adapt to a visible donor graft which will be apparent to others, endure a lengthy surgery and recovery which often requires relocating to the location of the transplant center and requires significant support from family members. Thus, the decision to proceed with transplantation requires a careful process of considering the implications of the transplantation alongside the potential benefits in improved function, return of sensation, and restored body integrity.

Options for patients with upper extremity limb loss include multiple surgeries and/or utilization of prosthesis. Unfortunately, prostheses are often a less than ideal solution. Patients with upper limb amputation are significantly less likely to use a prosthesis compared to patients with lower limb amputation, and those who wear an upper extremity prosthesis commonly do not choose to wear it every day or all day (5). Vascularized Composite Allotransplantation (VCA) provides individuals with upper extremity limb loss the possibility of transplantation of an eventually sensate, esthetically acceptable, and functional hand. However, VCA is a highly disruptive and burdensome intervention, with an initial lack of function, lengthy recovery, exposure to lifelong immunosuppressive medications, and increased risk of developing subsequent morbidities (6). In some cases, the outcomes of hand transplant have been devastating (7). The option of living with or without a prosthesis, carry none of these significant downsides and advances in prosthetic technologies continue to accrue (8).

Standardized outcome measurement instruments for VCA have not been developed and as yet we do not have quality of life research using validated scales comparing the outcomes of prosthetic use and upper extremity VCA (8, 9). In addition, in some instances, VCA recipients have been unable to sustain the complex medical regimens, threatening graft survival (10). For some recipients, subsequent re-amputation of the limb has occurred (11). Careful decision making on the part of patients, their care community, clinicians, and care teams is needed to avoid the tragic harms of ill-adopted grafts, for the benefits of VCA to be realized, or more appropriate approaches to limb loss to be continued or initiated. VCA represents high risk decision-making on the part of candidates and VCA clinicians (12). Little theory or guidance exists for how to support all parties in reaching a well thought and felt through VCA transplant decision. This article brings recent theoretical developments in shared decision making (SDM) forward that may be useful for supporting patients as they work through the different types of issues on which hand transplant decisions hinge. The diversity of issues in VCA is also used to extend and deepen the theory of Purposeful SDM more generally.

## 2 Background

In this paper we use the term “community” to refer those whose day-to-day life is affected by the patient's limb loss and treatments, along with other significant people who contribute to the patient's well-being through their relationship with the patient, direct caregiving, practical assistance, emotional support, or other interactions.

The road to hand transplant is long and demanding for patients and their community. It may begin from a chronic medical condition that has reached the need for amputation, congenital causes, amputation following trauma, or amputation after extensive multiple reconstructive surgeries. Most medical centers will not consider upper limb transplantation until the patient has had sufficient time to adapt, as best they can, to their limb loss. During the period following amputation and/or trauma recovery, and rehabilitation, patients will typically try and use several prostheses, each requiring learning, adjusting, and trialing. These trials require multiple, frequently demanding, time consuming, and potentially expensive interactions with healthcare, and often result in frustrations with prostheses that function inadequately (14, 15).

Some experts limit access to VCA to bilateral amputees, excluding patients with unilateral limb loss from transplantation as they consider that the risks of transplant-related immunosuppression exceed the benefits of VCA. Living without two functioning upper extremities places heavy burdens on patients and the community who assist patients with many activities of daily living. This dependance also takes a toll on the patient. Domestic and intimate relationships are heavily affected by limb loss, and the patient may find themselves encountering social stigma or unwelcome curiosity (16).

Once a patient approaches or is referred to a VCA center, they enter a process of extensive clinical evaluation that probes their medical suitability, motivations, psychological, physical, environmental, financial, social, caregiver, and financial resources; the presence of disqualifying behaviors (e.g., substance use, non-adherence to medications); adaptation to limb loss, the adequacy of their understanding of the risks and requirements of VCA; willingness to proceed, and a gestalt sense of the suitability of the patient for entering into a long-term relationship with the practice and its healthcare professionals. Multiple clinicians such as surgeons, transplant specialists, psychiatrists, physical medicine specialists, physical therapists, social workers are involved in the evaluation process, and the potential candidate and caregivers may meet with each of them individually several times over multiple visits occurring over a course of months or years (17).

At the conclusion of the evaluation process, the candidate may be deemed eligible, ineligible, or conditionally eligible for upper limb transplantation. Conditional eligibility may require the patient to take time to fulfil prerequisites established by the VCA team, including treatment for substance use, securing of financial resources, or psychological counselling.

The expectations, motivations, goals, and responsibilities of the patient, caregiver, and VCA team may or may not be well aligned in the evaluation period. The patient may enter with a strong desire to move quickly to surgery, while the care team fulfills their responsibility to ensure that transplant occurs only if transplant is medically advisable in the patient’s situation. For these patients, evaluation may seem a frustratingly long process of very limited value, while clinicians may feel that they must weather unreasonable expectations and demands. Patients may feel judged and try to appear adequate, compliant, and capable of managing and benefiting from transplant to sway clinicians’ judgement and eligibility decisions (18).

Evaluation however is not the only process that determines if a patient will proceed to transplant. Concurrent with, and subsequent to, evaluation, is a patient decision making process within which patients and their community work toward decisions regarding if hand transplant is the best path forward or if alternative approaches are preferable. In some respects, the evaluation process and the patient decision process are similar in that both conclude in treatment determining decisions. The processes differ however in significant ways, notably, the purpose of evaluation is to determine the medical likelihood of success, identifiable risk factors, and requirements that must be met before proceeding, all of which determine *suitability* and *eligibility*. In contrast, the patient decision making process has the purpose of establishing if the patient and their community want to proceed (or not), their criteria are more akin to *desirability* and *acceptability*.

Patients’ decisions for or against transplant may be difficult or relatively easy for them to make. However, the implications of the decision to proceed are such that the decision should not be made lightly. When difficult for patients, the weight of decision making can in itself be a cause of suffering or leave patients and their community frustrated, conflicted, or confused. Healthcare has a role to play to ensure that decisions are well considered, and that confused and struggling patients are not distressed under the weight of decision making. VCA care should include efforts to support and help patients in their decision making (19, 20). This supportive role is distinct from evaluation. Given the short and long-term risks, burdens, and disruptions of hand transplant, support also includes efforts to see if trouble with prostheses or living situations can be problem-solved in order to make less invasive approaches practical, acceptable, or worth trying.

Individual clinicians, care teams, and VCA practices have a dual responsibility–to ensure, through rigorous evaluation, that VCA will not expose the patient to unwarranted harm, and, to assist the patient in reaching the decision that is right for them, their life, person, and community. The former is, or can be made, a relatively explicit, formal, team-based, and procedural requirement with analogous models in solid organ transplant. The latter is more implicit, less standardized, and it is less apparent how to do it in the context of a new and rare intervention such as VCA, during which patients interact with multiple clinicians, frequently individually (rather than as a team), to discuss on multiple occasions a highly invasive elective surgery that has extensive long-term implications. While it is clear that evaluation happens, it is unclear how to support patients in decision making, and to what extent this occurs.

### 2.1 Shared decision making and its limits

Shared decision making is a patient-centered means of supporting patients facing both a challenging situation and a difficult decision, a situation that describes VCA decision making. In SDM, as it is traditionally conceived, clinicians invite patients to collaborate in decision making, share why a decision is needed, identify alternatives, describe their characteristic benefits and harms, elicit the patient's preferences regarding the options, and together with the patient agree on which alternative to follow (21). This model has been applied in many contexts including primary care, specialty care, and preventive care. Generally, SDM has been shown to increase patient involvement in decision making, knowledge about their condition and options, satisfaction with encounters, and to reduce decisional conflict (22). The practice of SDM and its body of evidence may be useful for helping patients to come to the right transplant decision for them.

Earlier work from our team (17, 18, 23) and other literature indicate that patients’ reasons for proceeding, or not, with VCA are multifaceted and motivated by different issues (16, 19, 24–26). Some patients, for instance, seek VCA treatment driven by a strong inner will to obtain it and for whom the substantial risks of surgery, rehabilitation, and lifelong immunosuppression hold little sway in decision making. Others who decide not to proceed are strongly influenced by these same risks, while others elect to forgo VCA because it is too difficult to put all the supporting logistical and caregiver structures into place or feel that they have adapted to limb loss to an extent where they feel that VCA is not warranted. Some make decisions based on where they are in their life's journey and if VCA has a home in the continuation of that story (17, 23).

These diverse justifications for VCA decisions stretch the capacities of conventional SDM models and their recognized limitations (27, 28). SDM to date has primarily focused on involving patients in decisions that are made over a fairly short period of time, and on “preference-sensitive” decisions conceived as made by weighing the risks and benefits of illness and treatments against preferences held by the patient. The criteria that different people give for their VCA decisions suggest that the balance of harm and benefits is only sometimes the primary justification for a decision and only one way in which conclusions may be reached. Additionally, VCA decision making may occur over a period of years, during which time the synthesis that the patient, their community, and care team hold of what is the best decision develops in substance, nuance, and quality.

Recently a new model of SDM, Purposeful SDM (28, 29), was developed spurred by the recognition that in medicine generally, the weighing of pros, cons, and preferences is not the only way in which patients and clinicians make well-formed decisions together (29). The Purposeful SDM model articulates that how shared decisions are made varies according to the problem that decision making is hoping to address and the situations that necessitate that decisions are made. For example, weighing pros and cons may be helpful when choosing a new antidepressant after the initial medicine proved ineffective, but the method of weighing is of limited value when the decisional issue is whether a patient is able to make peace with taking an antidepressant when for years he has viewed doing so as a sign of weakness. In the latter case, helping the patient explore, negotiate, and potentially reconcile his inner conflict may be the SDM method best suited to reaching a decision that responds to the issue as the patient experiences it. This paper develops Purposeful SDM for VCA and upper limb loss by drawing together and expanding on theoretical and practical accounts (13, 28–34) of Purposeful SDM and connects them to decision making in VCA and issues that patients, in their own voice, have grappled with in making VCA decisions. Box 1 provides an *at a glance* overview of Purposeful SDM for clinicians (18, 23).

**BOX 1 Purposeful SDM at a glance for clinicians**

**Patients’ and their communities’ decision making may be difficult and troubling due to:**

• The many beneficial and detrimental **effects** of VCA and its alternatives
• Conflicting **positions** within the patient or with others
• The life **situation** of the patient and the problems involved in making VCA or other options feasible, sustainable, and valuable
• The existential significance of limb loss and hand transplant for the patient's **being**

**These different issues require different SDM approaches**

• Identifying the **effects** of particular concern to the patient and helping them **weigh** them at a pace and depth that is productive and caring.
• Helping the patient and their community voice points of tension and **negotiate** conflicting **positions**. This may include creating alternative perspectives, exploring ways of accommodating different priorities and concerns, and fostering respect for people’s different positions.
• Facilitating and contributing to **problem solving** the unique challenges of the **situation**. This may include working with the patient to clarify the problems and opportunities of the situation, create new ideas, explore how barriers may be overcome, or how to make new ideas feasible and helpful.
• Help patients and communities find, tell, and reflect on their stories to **develop existential insight** into what VCA means for the patient's **being**.

Recognizing if a patient and their community is struggling with issues of effects, positions, situation, or being is critical to successful SDM and VCA care.

For more guidance see, Montori et al. Shared decision making as a method of care (13).

## 3 Purposeful SDM and VCA

Purposeful SDM is a problem-based model of SDM rather than the more common involvement models (21). Involvement models focus on advancing patients’ involvement in decision making and in determining the final decision based on their preferences and values. Purposeful SDM begins from the premise that decisions are made in healthcare to respond to the current or prospective problems that a patient is experiencing. Decision making is the process of figuring out how to respond to problematic situations, and SDM occurs when patients and clinicians work together in conversation to understand a problematic situation and form action that responds to it. Care and SDM have the same goal–to change the problematic situation by finding and instituting the way to change it. This makes SDM a method of care (13) that is part of the everyday practice, problems, and purpose of medicine rather than an ancillary effort to increase patient participation. Purposeful SDM presents different ways that patients and clinicians work through what to do in different types of problematic situations. The view of SDM presented in this paragraph aligns closely with the third mode of Purposeful SDM that will be described shortly, for this reason similar language and terms also appear there.

Purposeful SDM's theoretical and practical roots and themes arise in diverse places including philosophy (35), rhetoric (36–38), management (39), design (40–45), medicine (46), and clinical ethics (47) but the account above at its root most closely reflects the work of the pragmatist philosopher, John Dewey (35, 48). His two-part “question of action” (49, 50) describes the challenge that patients and clinicians grapple with in deciding what to do when things are somehow wrong and what to do is uncertain–the condition of much, if not most, of everyday medical practice (including VCA) and patients’ need for care.

Dewey's 2 part question of action (50):

*What is the situation that demands action? And, what is the action that the situation demands?*

Adapted to VCA this becomes

*What is the situation for which VCA may be an appropriate action? And, what is the action the situation demands which may or may not include VCA?*

**Figure 1:**
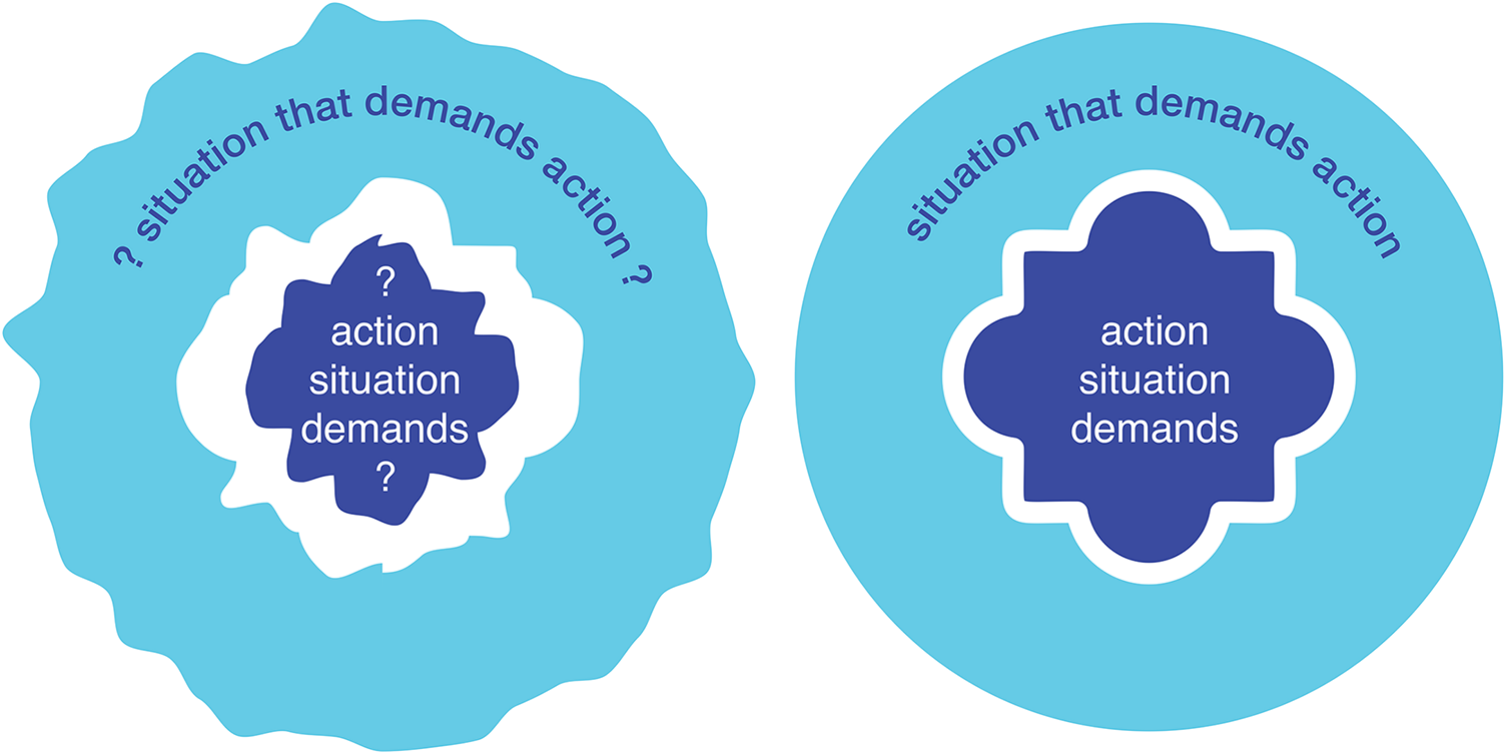
SDM as the shared endeavor to change a troubled and uncerta*in situ*ation (left) by working through how to change it (right).

Over the extended course of decision making, patients and their community and clinicians work to bring the two parts of the question of action into well thought, talked, and felt through alignment (Figure 1).

Dewey's concept of “problematic situation” is an important part of his work and its use in Purposeful SDM. A problematic situation is more than, and different to, the patient’s context, history, diagnoses, social determinants etc. as these in themselves are not inherently problematic, although they may be abstractions of experiences which are (46). For Dewey, a problematic situation is foremost experienced as having an emotional quality that may be troubling, dis-orientating, incapacitating, confused, uncertain, distressing, painful, tragic, confining, hopeless etc. (48). A problematic situation is what a patient and others are caught up in and struggling with. The limited means to change it is part of the struggles’ practical and emotional quality.

A situation, then, is active, not the background to decision making as “context” implies. The problematic situation is the struggling, not the arena. It is what people are struggling with and the struggle against it. Commonly, and clearly in VCA, a problematic situation is not one person's alone (e.g., a patient's) as various people are active participants in the struggle to change something in the real world. VCA care can be seen as care-teams joining a struggle and the support that they offer in decision making as changing the situation that the patient and their community are caught in.

Because a struggle is an effort to make an objective change, a situation is objective and is not locked away in a purely internal subjective feeling. The shared, real nature of problematic situations is reflected in language when we speak of shared experiences such as; a situation is tragic, or unclear, promising, urgent, distressing, restricting, hopeful, exhausting, stable, manageable etc. Each of these examples encompass objective conditions, an active struggle, and their shared emotional quality. In limb loss, particularly after adaptation, struggle may be too strong or acute a term to describe the situation, nevertheless, VCA is considered when an active response is looked for to something undesirable in a situation. In this context, the more general Purposeful SDM term endeavor, may be more appropriate than struggle.

Box 2 further summarizes the implications of Dewey's concept of a problematic situation for VCA and Purposeful SDM.

**BOX 2 Problematic situations and Purposeful SDM**

**A particular problematic situation includes:**

• The way it is experienced as difficult and confused.
• Its issues and the factors contributing to it.
• The possible ways of changing it–their recognition, availability, efficacy, limitations, configuration, feasibility, and any problems or opportunities they may introduce.
• What patients and clinicians prioritize or limit their efforts to addressing.

**This means that the situation changes as:**

• Possibilities and complications appear, come forward or recede throughout the course of decision making and care.
• Priorities or the scope of the problem shift.
• How it is experienced evolves.
• Factors contributing to it change.

From this perspective, decision making doesn't happen in a *context*, rather decision making continually changes the *situation* throughout its course, for example by coming up with a new possible way of simplifying arrangements for the surgical recovery period.

The appropriate method, purpose, and criteria for decision making changes alongside the nature of the situation.

Purposeful SDM connects types of situations, issues, methods, and purposes of collaborative decision making.

Purposeful SDM describes 4 modes of SDM, each concerned with problems of a specific type of situation and its particularly pertinent method of SDM.

The four methods and situations requiring SDM that patients and clinicians use to reach decisions together are:

1. Weighing pros, cons, and preferences when the effects of illness and treatments are worrying and uncertain.
2. Negotiation when intra or interpersonal positions are in conflict.
3. Problem solving when the frustrations of a situation thwarts day-to-day life and care.
4. Developing the existential insight required to act when life or being is fracturing or in existential transition.

The four methods are necessary because different types of problematic situations require that decisions are made in different ways.

Each mode of Purposeful SDM is organized around a central issue:

Effects
Positions (of people)
Situations
Being

**Figure 2:**
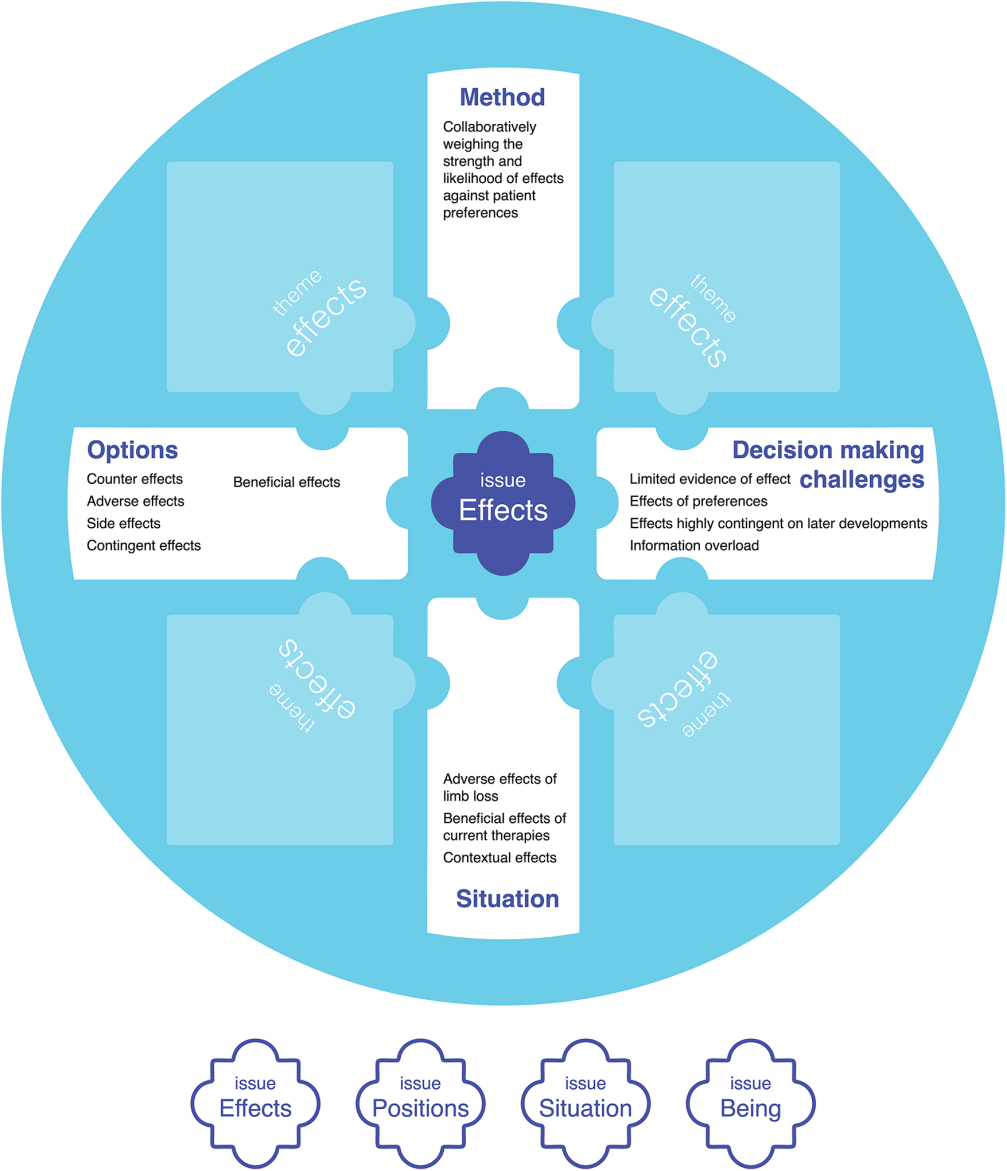
Central issues in SDM (*effects* partially developed as an example in this figure), that connect the situation being addressed, options for addressing the situation, why decision making is challenging, a suitable collaborative decision-making method, and the purpose of SDM and care. Table 1 presents an expanded form of this figure that includes all four issues.

Each issue is a common term, topic, and theme that connects different aspects of a mode of SDM, including the situation, method of SDM, possibilities for addressing it (options), challenges to SDM and care, and the purpose of SDM and care (Figure 2).

**Table 1:** The four central issues of purposeful SDM in the context of VCA.

Kind of issue	Situation presents or is interpreted as	VCA as an intervention is	Issues rendering decision making challenging include	Purpose and method of SDM
Effects	The combined adverse or beneficial effects attributable to limb loss, comorbidities, treatments, psycho social determinants, and contextual forces-historical, current, short-term, long-term, and contingent. The overall character is the situation is one of uncertainty.	A counter effect with relatively likely (e.g., symptoms of rejection) or less likely associated beneficial and harmful effects that are more-or-less independent, manageable, and detectable with medical, functional, psychological, and contextual consequences. “Outcomes” generally refer to effects, and consideration of VCA is predominantly outcomes led.	The quantity, diversity, uncertainty, and interaction of potential causes and effects. Limited patient reported outcomes/effects (PROS) and patient-important effects. The effect of patient preferences on how to value and balance conceivable positive and negative effects. The exponential number of trajectories that arise from tracing prospective contingencies. Limits of human cognition to comprehend, anticipate, plan for, and grasp the significance of contingent effects. Low quality evidence of effects. Expert knowledge of effects bounded in sub-specialties.—Limitations of patients, caregivers, and specialties to understand differing expertise.	To collaboratively weigh and balance, to the extent possible, contingent medical and patient-important effects in light of patient preferences and values. To enable patients, caregivers, care team members to discover, understand, and utilize knowledge of diverse effects in making judgements. To support patients in living with uncertainty/anxiety and working with unknowns throughout the extended decision making process.
Positions	Perspectives, points of view, opinions, motives, priorities, stake, goals, expectations, hopes, bonds between, obligations to, or wishes of the patient, community, or care team are in tension or conflict with another. This conflict may be within or between persons or groups. The character is one of reliance on and commitments to others, distributed autonomy, not/knowing or not/getting what one or others want,or are willing and non-coercively able to do.	The exercise of individual and group desires to make a change and attraction to VCA as means of making that change. “Convinced or not convinced” refers to the relative positions that parties to the decision adopt in relation to one another and VCA decision making is achieved through convincing or persuading oneself or others to proceed or not.	The positions of people may be ambiguous or unknown to themselves or others. Positions may be stubbornly fixed, making agreement or the resolution of conflict impossible. The interests of different parties may be diametrically opposed. Positions may be fueled by blind hope, hopelessness, guilt, shame, fear, or passivity. People are invested in their positions, making conflict emotional or possibly heated. This may lead to the breakdown of the communication needed to work through conflict. Positions may appear irrational to others. This may lead some to consider that the person is irrational or does not care.—Intrapersonal conflict may be mislocated in or create interpersonal conflict.—Positions may be in flux, changing from interaction to interaction because of circumstance, the influence of others including media, language use, changing perspectives, deal-making, pleas etc. This may make parties feel that the ground keeps shifting or plague individuals or groups with paralyzing doubt or prompt adamantly latching on to the security of a firm decision or position prematurely.—From their positions, people may be talking past each other, or using similar words but not talking about the same thing.—Power relations may inhibit the expression or authenticity of positions.	To give voice to, respect, distinguish, challenge, negotiate, create alternative, reconcile, accommodate, and amplify individual and collective wishes, commitments, and intentions.
Situation	A way of life and living, more-or-less adapted to limb loss with experienced particular qualities, social relationships, activities, functions, roles, potentialities, demands, aesthetics, rhythm, challenges, joys, resources, values, constraints etc. For the purposes of VCA, bounded by the experienced and potentially modifiable needs of the patient and community that are determined by multiple contributing interacting practical, medical, and emotional factors, the potentialities available, the capacity to use them to recompose the situation, and the nature of what's (un)desirable or valued in the current situation and in the changing way of life being created.	A reconfiguration of ways of life and what it is possible to be and do within them. “Function” refers to a component's ability to contribute to, integrate with, and be sustained by, a whole of which it is a part. For VCA to be successful pre- and post-surgery, it must function in a person's life, enable other aspects to function in new or enhanced ways, establish functional ways of managing and recovering from the problems it brings, and meaningfully change how life is lived. VCA decision making proceeds by resolving and coordinating how to make VCA possible (or not), why the change it brings to the patient's and their communities, life, is valuable, and actualizing other ways of changing the situation if they are more appropriate.	The multiplicity of factors that shape the way that life is lived. The contribution, desirable or undesirable, that factors contribute individually and in complex interaction. The partly confused nature of the situation, including its troubles and means of addressing, and significance. The particularity of the situation makes it only somewhat comparable to others. That ways of living are inherently social and not isolatable to events affecting, or behaviors of, a disconnected individual. The holistic nature of change. The coordination and adaptation necessary to compose resources, processes, people, capabilities, purposes etc.—The dynamic form of the situation.	Method of SDM: Problem resolution. By working through problems together, patients, their community, and clinicians are able to judge if VCA can be made to be a feasible and desirable way to modify the patient's life. Involves envisioning how life will be at different points of the VCA journey, including continuing immunotherapy and other life altering factors. Purpose of SDM To re-form/re-compose how life is lived by shaping action that is responsive to its problems and potentialities. To contribute in concert with patients and their community in this shared and mutually-enabling endeavor. To discover in how life is lived the situation that requires action, and the action that it requires and to compose its many facets into a course of action that makes practical, intellectual, emotional, and spiritual sense, and which is feasible, sustainable, and adaptable.—To continue to reevaluate and addresses the changing situation, action taken, and problems encountered.
Being	The patient and their community's relationship to what brings meaning or purpose to their life has dis-integrated by circumstance or is re-integrating in existential transition, straining the bonds that bind them together, the sense of self that they hold, and questions what gives meaning in life when old ways of being appear impossible and old narratives struggle to make meaning of change. Certainties have disintegrated taking meaning with it, and the reconstruction of meaning is more-or-less partially complete.	A new beginning and the restoration of self and what makes it whole. A way to integrate the past with the present and future.	The impossibility of seeing oneself or others in an all-inclusive holistic. The limits of words to encompass existential meaning. The stripping of existential significance from the language, facts, voice, listening, and practices of contemporary care and larger social life. The necessity of relying on imagery, partial accounts, memory, myths, and story to reveal meaning that is not expressible in simple facts, chronologies, or affect. Fissures between different systems of meaning. Professional distancing along with other isolation of selves that blocks participation in finding the meaning that would validate VCA.	Method of SDM: Integration and insight development. Purpose of SDM: By using dialog, narrative, metaphor, questioning, reflection, and insight development to understand who people (patient and communities) are currently and historically and discern a meaning that allows for the trauma of the past, the diminishments and joys of the present, and the possibilities and impossibilities of an emerging future to achieve harmony. Through this process, the existential significance of decision making becomes clear as does the decision of whether to bring a course of action (e.g., VCA) into the life of the patient and their community.

These issues, developed through theory in the social decision making disciplines of Rhetoric (36–38) and Design (40–45), reflect different types of struggles that lead people to consider VCA and the endeavor of working through them in decision making and care. Sometimes, for example, the struggle is with the *effects* of limb loss, the limited effectiveness of prosthetics tried, and with the negative effects and uncertain outcomes that VCA would introduce. Sometimes the struggle is with the life *situation* of the patient and whether it's possible to put together the necessary resources, time, logistics, and people needed to make VCA or alternatives feasible or a realistic option. Collaborative problem-solving tries to figure out if and how VCA, or other options, can be made feasible and at what costs, while collaborative weighing of effects tries to determine if uncertain benefits are favored over what's known of uncertain harms. A qualitative chart review of 9 patients evaluated for VCA found that their motivations for considering hand transplant were traceable to these four issues (17).

Each of the four issues are heavily implicated in VCA decision making and in the use of SDM to help patients reach a well thought, talked, and felt through life changing decision. Effects, positions, situations, and meaning are implicated in SDM for VCA because they reflect the range of struggles that are the imperative of patients’ decision making, and because arguably, each must be appropriately thought, talked, and felt through to come to a well-grounded conclusion.

### 3.1 The four central issues of purposeful SDM

Table 1 expands upon each of the central issues described in this section.

#### 3.1.1 Effects and their weighing through SDM

The effect of the events that led to limb loss—bodily, psychologically, and socially, and the ongoing effect of living in the absence of one or more limbs establish reasons to consider VCA. VCA, has several potential beneficial effects for quality of life, function, appearance, and psychological well-being. Additionally, VCA comes with many potential side effects including potential graft rejection, immunosuppressive related cancers, infections, and harms to other organs, strains on mental health and relationships. Further, VCA has significant effects on life post-transplant including lengthy and immobilized recovery followed by extensive rehabilitation, close medical supervision, lifestyle modifications. How significant these matters are is affected by the patient’s priorities, goals, and preferences. These matters cannot be overlooked. Care teams have an obligation to discuss them—at a minimum for purposes of informed consent [which is fundamentally different to SDM (51)] and candidacy evaluation. But more than this, the seriousness of the potential adverse consequences of VCA mean that even if the patient doesn’t make their final decision primarily on the balance of pros and cons, their consideration is required, otherwise the integrity and trustworthiness of their decision is questionable. Further, possible adverse effects or events should not only be known and weighed, they should also be planned for and managed, requiring patients and caregivers to take preparatory actions such as having plans in place to handle the almost inevitable rejection episodes.

*I mean I don’t know much about this immunosuppressant-type drugs or anything like that; but if you have to take them for the rest of your life, what happens if you can’t take them for the rest of your life? Does your hand fall off?” (female, 67, gunshot accident as a child)* (23).

A SDM conversation arising out of this patient's question would work through what is known about the effects of stopping immunosuppression, how it would be best handled medically (amputation of the hand rather than it falling off) and how subsequent care would be managed, the sort of circumstances that would have the effect of leading to a withdrawal of immunosuppression, and their likelihood. This conversation would create an ability to weigh this issue alongside other beneficial or harmful effects.

#### 3.1.2 Positions and their negotiation through SDM

Determining the balance of preferred effects is in itself inadequate for reaching a well-rounded VCA decision, neither are the uncertain effects of VCA the only reason why the decision is hard.

Many people are affected by an individual's limb loss, and many people will be affected by the decision making process and the eventual decision to proceed with transplantation or not. This means that the decision is not simply the patient's to make. Although consent is ultimately the patient's alone to give, the decision is shared, to a greater or lesser extent, by members of their community and the care team. Typically, a designated committed caregiver must be identified in order for a patient to be eligible for VCA. Even, when others are not explicitly identified as co-decision makers, their position with regard to transplantation and other options is an important consideration because their attitudes and willingness to affirm and support the patient may affect the commencement of, success of recovery from, and life after, transplantation.

The positions of the various parties implicated in VCA may be in conflict, for example when a patient is motivated by a desire to right the wrong that a careless accident inflicted on their family, while a spouse who bore a significant burden of the trauma and recovery does not want to put themself and their relationship through that again with transplant. Potential benefits and harms are not the primary issue here and weighing them may do little to help patient and spouse reconcile their positions to the extent that a respectful decision can be made. Similarly, a patient who enters evaluation determined to receive a transplant may have expectations and an agenda that is in conflict with the agenda of members of the care team. Negotiation is a method of SDM suitable for situations of conflicting positions.

Additionally, the patient may be conflicted within themself. For example, when the appeal of being a part of cutting-edge medicine may be strong, while the stories of people who have had bad VCA experiences tug at the patient. A decision to proceed or not may come down to which of these positions hold, or if an internal reconciliation can be negotiated.

Inter or intra-personal conflicts are looked for as part of psychological and social evaluation processes. Beyond evaluation, SDM requires the care team to appropriately support the patient and those around them to identify, express, and reconcile conflicts. The decision may not be effectively made if positions cannot be successfully brought forward and negotiated, and the quality of a decision reached depends in part on the relationship of differing points of view, expectations, hopes and fears, and commitments.

*…it takes me back to when people would come to the bedside to talk to me about, ‘Wanna transplant this, wanna transplant that, and we can take your thumb, and we can take your fingers, and we can take ligaments from the other side,’ you know all these conversations. And anybody else that came into the room was speaking the same language as they were. And I wanted to hear an advocate for the prosthetics, when that was a possible action, and an advocate for all the other options, not somebody who was presenting [all] the options under the umbrella of the one choice*…*” (male, 71, arm shot off in active duty 15 years prior and reattached)* (23).

In this instance, the participant is describing a lack of the perspectives/positions of advocates of prosthetics and other options that would supplement the positions of different members of the care team that have been articulated in a singular voice. These multiple perspectives and voices are needed so that various positions are available to set next to others to determine if and where any conflicts lie, how he feels about them, and so that he is equipped to reach a decision by negotiating his position in relation to those illuminated by others.

#### 3.1.3 Situations and their problem-solving their limitations through SDM

Limb loss changes how people, the patient and those alongside them, live their lives. It restructures roles, routines, responsibilities, resource needs, work activities, activities of daily living, hobbies, social interactions etc. Adaptation is common along with a lot of very practical problem-solving and reorganization. Life evolves with changes in function, routines stabilize, problems are solved, new technologies become available, new problems and limitations develop, children grow or leave home, jobs and housing change etc. A life situation may work well in some respects while also enduring, incorporating, or working around its practical barriers and limitations. Considering VCA may be sparked and constrained by situations arising from day-to-day life.

A key issue is how VCA will change the living situation and whether the disruption that it may introduce in the short, medium, or long term is warranted. The issue of situations grapples with how life is now, what it could be like in the future, what will it be like getting there, and how best to get there. A SDM method of problem-solving digs into if there is a need for VCA, what VCA should accomplish, and are there other more suitable ways of addressing current needs.

If VCA appears like a promising way of making changes to the living situation, then working out everything that would need to be put in place and done so that the lead up to transplant, surgery, and its extended recovery and rehabilitation are feasible, sustainable, and tolerable is necessary. Only so much can be anticipated, but practical challenges such as insurance coverage, time off work for patient and caregivers, living close to the medical center during recovery, childcare coverage, and competing responsibilities quickly emerge. SDM and family decision making utilizes collaborative problem-solving to determine if all the factors can be aligned in such a way that VCA is feasible and manageable. Imagining and trying on in supportive conversation if there are logistical configurations that may work as well as taking concrete action to see if problems can be resolved–for example starting down the road of insurance pre-authorization–are all variations of collaborative decision making through problem-solving. As problems in the situation are anticipated, planned for, and are or are not overcome, the decision of whether to proceed with VCA in the patient's situation becomes clearer.

Purposeful problem-solving may also prompt new ideas for what to do or how to get around problems. For example, modifying an occupational therapy program, weight loss to improve function or the ability of the body to undergo and rehabilitate from VCA surgery, getting a pet as a counter to isolation, changing from virtual to physical work location, creating a fundraising website to make treatment more affordable, moving to another town, etc. All of these possibilities modify the life situation of the patient and their community and what makes it feasible, sustainable, hopeful, and meaningful.

*I’ve gotten so used to living without my arm, my prosthesis almost gets in the way. It almost gets in the way because I’ve learned how to do so much in my armpit and holding my stump down and all kinds of stuff*… *I’ve learned how to do stuff so much without my arm, now my arm is like–I’m learning how to re-use my arm and keep it out of the way. When I need it, I need it. I haven’t needed it for nothing yet.” (male, 59, skin cancer led to arm amputation one year prior)* (23).

Here the patient is describing that his prosthesis hasn’t integrated into his day-to-day life and that it isn't supportive in his situation. A SDM conversation would probe if there are problems in how the patient and community live their life. If there are, it would use a conversational back-and-forth to see if there are other ways of addressing them than VCA, imagining what life would be like if they are adopted, and problem-solving to see how their limitations might be overcome. When turning to discuss VCA, the practicalities and worth of other approaches examined allow for comparison with VCA. Discussion of how VCA might change needs in the situation examine if VCA is responsive to the situation. Further discussion of how life would be throughout the lead up to and rehabilitation from surgery bring forward problems to be solved that inform decision making. Through these discussions, care team members may be able to help the patient with any situational issues that they are currently experiencing.

*“I mean when you’ve been through the rejections stuff and, basically, the therapy and how long it would take to recover, that was probably the biggest thing for me, where they’re talking, and you could be down for a year just trying to let it heal and rehabilitate. I have a young daughter. I’m not going to be out of anything for a year. That can’t happen. I mean I’ve got to be there for her, and that’s the #1 thing I’m going to do, and I can’t walk away from my farm” (male, 49, congenital hand, add’l wrist amputation 2 years prior due to cancer)* (23).

In this instance, the young daughter figures strongly in the patient's situation. Envisioning going through VCA highlights the patient’s need to be there for his daughter. Unless problem-solving conversations can create a way in which this is possible, the decision of whether to look further into VCA may essentially be made.

#### 3.1.4 Being and developing existential insight through SDM

Traumatic, or other, limb loss may shatter the identity of patients and families and the story of their lives individually and collectively. This is an existential splintering in which the meaning of lives and how they are understood fracture and more-or-less successfully transition to new ways or states of being. Experienced this way, VCA has existential significance, perhaps offering the possibility of integrating fragments of being into a restored reality or storyline of who people are and can be. Here, VCA is in a sense, a restoration of wholeness that goes beyond anatomical or functional completeness.

*“They [VCA recipients] don’t want to [only] get their hand back so that they can go back to work, they want their hand back so that they can touch the face of their loved one. So that they can hold the hand of their loved one*…*it’s holding hands and feeling the skin of their hands. [The hand] kind of is an intimacy organ, you could say*… *they sustain these primary family relationships. So after I started having these new thoughts [of hands as intimacy organs] I started thinking ‘now I really haven’t thought this through the right way.” (VCA team member)* (18).

In this excerpt, a member of a VCA care team reflects on the insight that they have developed into the existential significance of limb loss and hand transplant. The clinician has moved their thinking from a clinical understanding of the positive and negative effects of VCA or patients’ and their communities’ situations toward the deep existential restoration that VCA offers in the places that deeply matter to people. Issues of being lead SDM conversations towards grasping the human significance of what has been lost through limb loss, and what may be made whole again, perhaps in new ways, through VCA or other options. These conversations seek for the meaningful, and meaning is often made present through the evocations of images and story and medical facts and evidence may be so dry and devoid of heart as to make them mute and largely meaningless. In the clinician's telling, the image of holding hands alone is sufficient to develop insight into the core being of patients and their community. Further SDM conversations and stories around the issue of being reveal more glimpses of meaning and over time they may coalesce into a deep existential sense of if VCA is right and good. That sense may make the pursuit of VCA, or not, self-evident, not only to the patient and their community, but also to the clinicians who through participation come to deeply know the significance of the care they offer and the home that they have in the lives they've entered.

### 3.2 Maintaining heterogeneity

Purposeful SDM is a method of care in which patients and clinicians work through how to change the patient's problematic situation. Each heterogeneous issue is a place in which life with limb loss and thinking, talking, and feeling through what to do may be a struggle. The four issues, effects, positions, situations, and being are heterogeneous because they differ in kind as well as in substance. They are real in their own way, but they are not real in the same way. Positions held within or between people that are in conflict, are not effects differing in likelihood, magnitude, direction, and preferability, for example. Treating limb loss and its necessary SDM through heterogenous issues, allows the substance of multiple struggles to be grappled with as the kind of issue that they are and to find good reasons to proceed with hand transplant, or not, that are grounded in each patient's and their community's body, person, life, and being.

Studies employing qualitative methods (16, 19, 23, 52) have identified many very important material themes associated with patients’ VCA decision making including; independence, intimate relationships, treatment adherence, care center's experience, success rate, aging with transplant, publicity, and identity. Purposeful SDM does not distinguish specific material themes associated with limb loss or any other condition, rather its four issues can be used to locate the ways in which material themes are problematic for an individual patient. For example, the following quotes from patients in differing studies touch on themes of appearance.

1. *“…it might make it easier on my family if I didn’t stand out in the crowd. That’s the problem. People don’t forget you, you forget them but, oh yeah, you’re the one-armed person and you know they’ll think that in their head but won’t say it”* (16)
2. *“It has always been important to me to have new real hands and not plastic or silicone hands.” UE. transplant recipient* (53)
3. *“Uh, me personally, I just couldn’t get past the portion of wearing-in my head, I like look like at it as wearing somebody else’s arm, like a cadaver arm”* (26)

In the first instance, the issue of appearance is presented as an issue of situation–the encountering of other people alongside family in social situations. In the second, the issue is the patient's position with respect to real and plastic hands. In the third case, the issue is existential–the integration of another person's arm in the patient's identity and being.

Locating problematic aspects of appearance as a specific type of issue allows the clinician to explore problems of appearance in patient-appropriate terms. For instance, if patient 2 were ineligible for transplant, discussion may support the patient in the grief of having to let go of a strongly held position, and when the time is right turn towards exploring ways of making a prosthesis most aesthetically acceptable.

Failing to locate the issue can lead to unsupportive care. For example, describing in detail to patient 3 how carefully the care team may work to find the closest aesthetic match of donor to the patient when the patient is struggling with if they can live with themself with another person's limb is tone deaf and may diminish trust. Allowing material themes or information to be heterogeneous in their issues is necessary to meet patients and their community where they need support.

Traditional models of SDM accommodate some heterogeneity, predominantly by attempting to transform heterogeneous issues into a homogeneous preferred effect frame in which positive and negative effects can be weighed as preferences. There are limits to this approach, however. For instance, speaking about the likelihood and extent of restoration of touch as an “effect” associated with VCA leaves out the existential significance of holding a child's hand, or how touch for a pianist extends performance beyond accurate and reliable motor function to an intimacy with the instrument that is felt in the music created and in the distinctive touch that the artist has for the piano. What is desirable and meaningful in these examples is not simply a “preference”, nor are they advantageous “effects” of VCA, contextual “effects” that affect the decision, or a designated goal of VCA at which the “effects” of VCA are aimed.

Specialists in the care team are disciplinarily oriented to different kinds of issues e.g., situations in social work, conflict or being in psychiatry, effects in pharmacy etc. Patients and their community, however, carry multiple issues with them into individual consultations. VCA clinicians require some facility with working with heterogeneous issues so that they can assist the patient to the extent that they are able, to refer patients to other professionals equipped to help, to develop a holistic appreciation of what the patient is experiencing and to share a common understanding with other care team members, and importantly so they do not coerce issues as they are experienced by the patient into the kind with which they are most disciplinarily comfortable. Doing so risks not acknowledging the heart of the matter, leaving the patient unheard and not cared for.

Similarly, some members in a patient's community may be implicated with particular issue types. For example, practical issues constraining a patient's situation are a likely concern for direct caregivers who must work with and around them. Many will have used problem-solving methods of SDM with the patient and others over the long course of recovery from limb loss and will help tackle the practical issues that need attention in order to put a plan that includes VCA in place. Their problem-solving work focused on feasibility, significantly contributes to SDM and the final decision. SDM between patients and their communities around this and other issues in large part happens outside of clinical encounters and may develop over an extended period of time. There is the opportunity for clinicians to enable and support these extra-clinical SDM conversations, perhaps by developing and sharing useful resources or approaches for talking through differing issues.

Resources such as these based on Purposeful SDM have been explored, for instance, in the development of a prototype online conversation aid intended to help patients, their community, and multiple clinicians over time to think, talk, and feel their way through hand transplant decisions and the patient's life with limb loss. Supplementary Material shows screenshots from different sections of this conversation aid intended to support conversation and extended reflection on issues of effects, positions, situations, and being.

### 3.3 Implications for outcomes, practice, and adoption

Clinically and in most research “outcomes” are used to indicate and report success. Outcomes as currently conceived are effects, and effects are adopted by diverse disciplines as a singular orientation that facilitates communication and multi-dimensional evaluation. When what has meaningfully and significantly changed through limb loss or hand transplant occurs in issues of positions, situations, or being, conversion from these themes to “effects” will inevitably obscure, miss, or distort what has happened. For example, quality of life (QoL), as an effect, does not describe how VCA has changed a patient's unique day-to-day experience and way of living (54), nor do functional outcomes, such as grasp strength, reveal how the action of grasping concretely changes how life is lived, nor does non-adherence to physical therapy as an outcome describe the realities of the life that treatments, for good reasons, struggle to adhere to (25, 55, 56).

Similarly, satisfaction as an effect, may be a distorted conversion of the conflicts that patients experienced around VCA, new tensions that have arisen, and the success of negotiating them. Satisfaction doesn't describe those tensions, if and how they have or are being negotiated, and what the reconciliations are. In the case of issues of being, the touch that is in the music of a pianist and what that touch means in the being of a recipient resists being characterized as an effect. In these three instances, VCA is associated with meaningful change, but understanding those changes requires considering them in terms of the kind of issue that they are. If attempts are made to convert them to effects, careful attention should be paid to what is lost in translation, and the significance of that loss for the prudent development of models of care.

**Figure 3:**
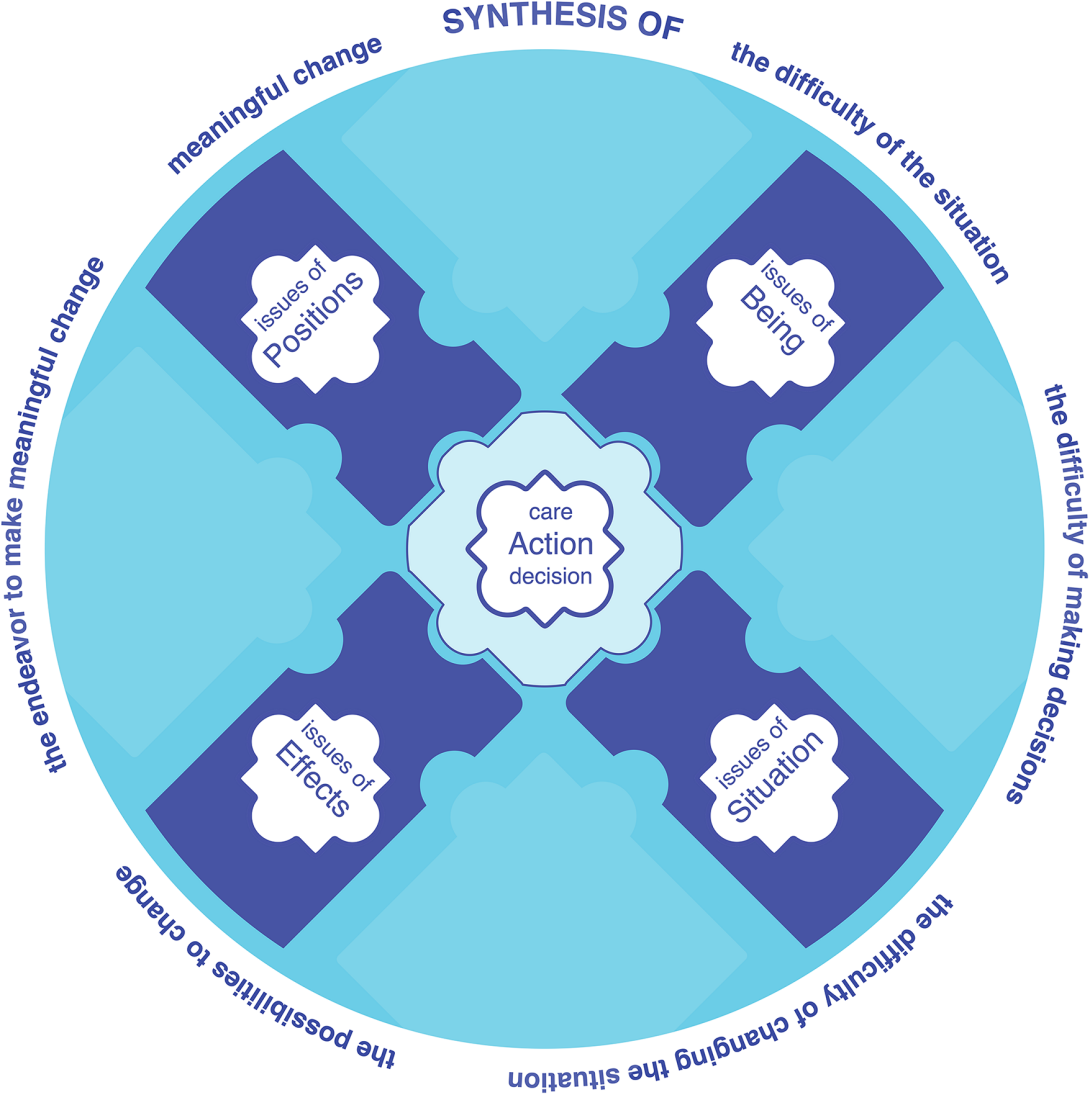
The synthetic shared purpose of care and SDM.

In uniting the difficulty of the situation–with the difficulty of making decisions–with the difficulty of changing the situation–with the possibilities to change–with the endeavor to make meaningful change–with meaningful change, Purposeful SDM and care share a common purpose–to act well in the situation that the patient is caught in Figure 3. This close connection also means that supporting patients in difficult decision making is an organic part of caring for the patient in their difficult situation.

The extended dialog between patients, communities, clinicians in homes and in clinics creates and strengthens the ability to decide, the capacity to support each other, and reveals the deep reasons for, meaning of, and value found in what is decided upon (50). When done purposefully, these conversations are SDM. The dialog not only allows final decisions to be made about what do about a problematic situation, the dialog itself changes the situation as part of its process. What is fraught shifts as options emerge or recede, what it is desirable to change becomes of greater or lesser priority as effects are weighed, what was unbearable may become bearable, what was assumed to be straightforward may become complicated, shifts in positions open and close doors and brings people into and out of care commitments, the practical limitations of the situation change as they are problem-solved, and as existential insight develops the bonds between patient, clinicians, and community deepen and the foundations for shared action come into being. The dialog of SDM and care changes a problematic situation from one that was initially paralyzed into one that is ready to accept, sustain, adapt to, and realize the human value of the action decided upon (Figure 1).

Enhancing clinicians’ abilities to care for people through issues of conflict, situations, and being may greatly enrich patients’ lives. Not only does it help ensure the ongoing success of transplant and strengthen the abilities of patients and care teams to manage post-transplant challenges, it also means that the patient feels cared for in their pre-transplant reality of living with limb loss. Trust is earned. Trust forms when clinicians help patients with the issues that they are struggling with. Clinicians similarly trust patients as they grapple with challenges together. Trust is critical to the commitment that patients and care teams make to one another over the long haul of getting to transplant and the lifelong care it requires.

There are other related decisions where Purposeful SDM may be helpful. For example, families that are asked if they would consider donating a deceased family member's hand also need support and grapple with issues of effects, positions, situations, and being (57, 58) in difficult circumstances. Problematic situations motivating other VCA transplant decisions, e.g., face, uterine, or penile, are also heterogeneous in their issues, as are issues in the much more common solid organ transplant setting. Purposeful SDM has not yet been explored in these areas. This approach to SDM may be particularly helpful in face transplant as the face is frequently core to a person's being, and the face limits and expands what is possible and experienced in the deeply social situations in which people live and thrive.

There are challenges to the adoption of Purposeful SDM by clinicians and transplant programs. It requires de-emphasizing evaluation without sacrificing its rigor and function. It favors not abandoning patients and opening care pathways or smooth and meaningful referrals for those who are not eligible for transplant, and it promotes broadening commitments to patients beyond technical expertise. As has been emphasized throughout this article, Purposeful SDM in hand transplant challenges the assumption that it is exclusively the effects of hand transplant that are the primary motivations for and against VCA. We have further suggested that outcome effects can only offer a partial accounting of the success or failure of VCA at the patient, practice, or scientific level. This does not mean however, that better clinical and patient important effect measures should not be developed and used to improve care. Qualitative or critical, social science, nursing, or humanities’ methods may further help understand the significance of change in issues of positions, situations, and being. While welcomed and necessary, it should be recognized though that deploying constrained qualitative methods to report “subjective effects” will still not get at the heart of issues of positions, situations, and being. In patient care, practice improvement, patient engagement, and research programming the voices of those who speak of the experience of positions in conflict, situations in jeopardy, and being in its humanity should be listened for, learned from, and valued in their own terms.

In spite of these challenges, the successful adoption of Purposeful SDM is greatly enabled in three ways. Firstly, in their own lives and in their interaction with patients, clinicians recognize the presence and importance of opposing effects, conflicting positions, problematic situations, and existential being. Secondly, clinicians already use methods of weighing effects, negotiating positions, problem solving situations, and developing existential insight. And, thirdly, because issues of effects, positions, situations, and being permeate life and medicine, Purposeful SDM is not just one more thing that busy clinicians are required to do, rather Purposeful SDM is the amplification and extension of understanding that they already have, methods they already use, and purposes already inherent in the practice of care.

In response to the complexity of VCA, this manuscript has articulated in greater detail and in new aspects the theory of Purposeful SDM generally and adds significantly to the SDM literature. Specifically, the fuller account of problematic situations is new as is distinguishing and organizing Purposeful SDM in terms of heterogeneous issues in contrast to deliberative methods, or kinds of situations as previously developed (13, 28–32).

## 4 Conclusion

The medicine, science, and potential success and benefits of VCA for patients continues to develop. Treatment determining decision making however remains difficult. The life-altering and life-threatening implications of hand transplant demands rigorous programs of evaluation. These implications and the problematic situations of those seeking care also makes patients’ decision making intellectually, practically, and emotionally fraught. Separate from evaluation, patients and their communities need support in making decisions throughout their VCA care journey. Purposeful shared decision making may help clinicians provide this support, ensure that patients are able to make decisions that are right for them, build the trust and caring that will allow patients and care teams to weather struggles along the way, develop common understanding and coordination within the team, set hand transplant or other approaches up for long term success by grappling with preferences, understanding and accepting risks, aligning expectations, reconciling conflicts, creating plans that are practically feasible, sustainable, and adaptable, and honoring the humanity of all those–clinicians, communities, and patients participating in life-altering and life-affirming care.

## Data Availability

The original contributions presented in the study are included in the article/**Supplementary Material**, further inquiries can be directed to the corresponding author.
