# Supplementary material for: The role of the purposeful shared decision making model in vascularized composite allotransplantation
Source: Front Transplant. 2024 Jul 9;3:1421154. doi: 10.3389/frtra.2024.1421154 (PMC11235292; doi:10.3389/frtra.2024.1421154)
Supplement: Supplementary file 1 [file Datasheet1.pdf]

frontiers

Supplementary Material

The Role of Purposeful Shared Decision Making in Vascularized Composite Allotransplantation VCA

Ian G. Hargraves*, Kasey R. Boehmer, Hatem Amer, Cassie C. Kennedy, Joan M. Griffin , Dawn M. Finnie, Victor M. Montori, Fantley Clay Smither, Samir Mardini, Steven Moran, Sheila Jowsey-Gregoire

* **Correspondence:** Corresponding Author: hargraves.ian@mayo.edu

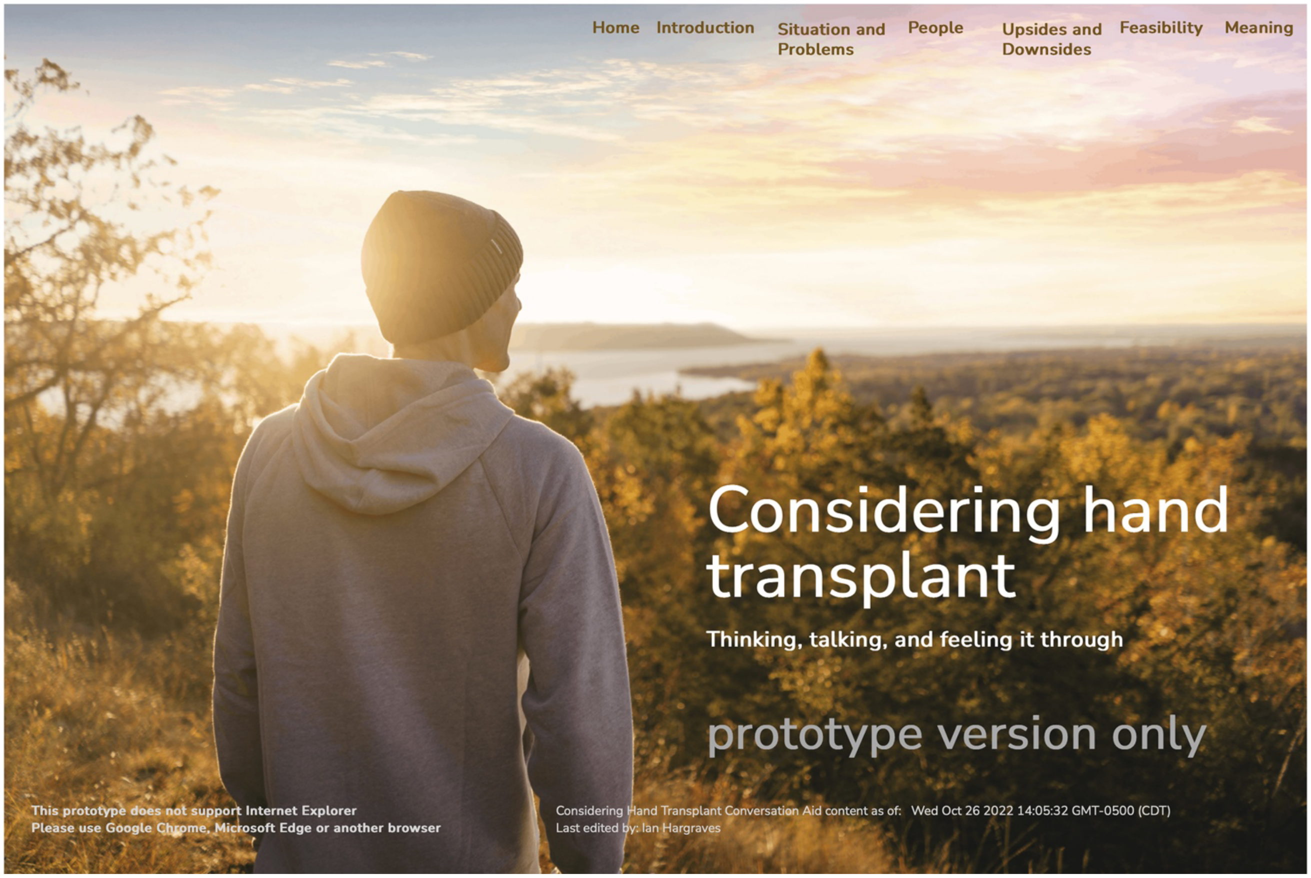

Supplementary Figure 1. Screen from a prototype online conversation aid to assist patients, their community, and clinicians discuss, reflect on, and make decisions regarding limb loss and hand transplant

Hand transplant is a big step

There are several ways to help with limb loss, hand transplant may or may not be the best approach for you.

This guide will help you, your care team, and those close to you think through and discuss all the issues that need to be considered.

Deciding wether to proceed with hand transplant or not will take time.

Over the weeks, months, or years that you, your care team, and those close to you are making this decision, you can return to this guide for information and to help you think through your decision.

This is your personal guide, in it you can record information, your thoughts, concerns, and plans. You may or may not wish to share it with others.

As this is only a prototype version, please remember to save any changes that you have made to guide before closing this window and load the most recent content into the tool when next using it.

Supplementary Figure 2. Introduction to the prototype conversation aid. Each patient has their own conversation aid to which they can add and edit content over time, creating a "living document."

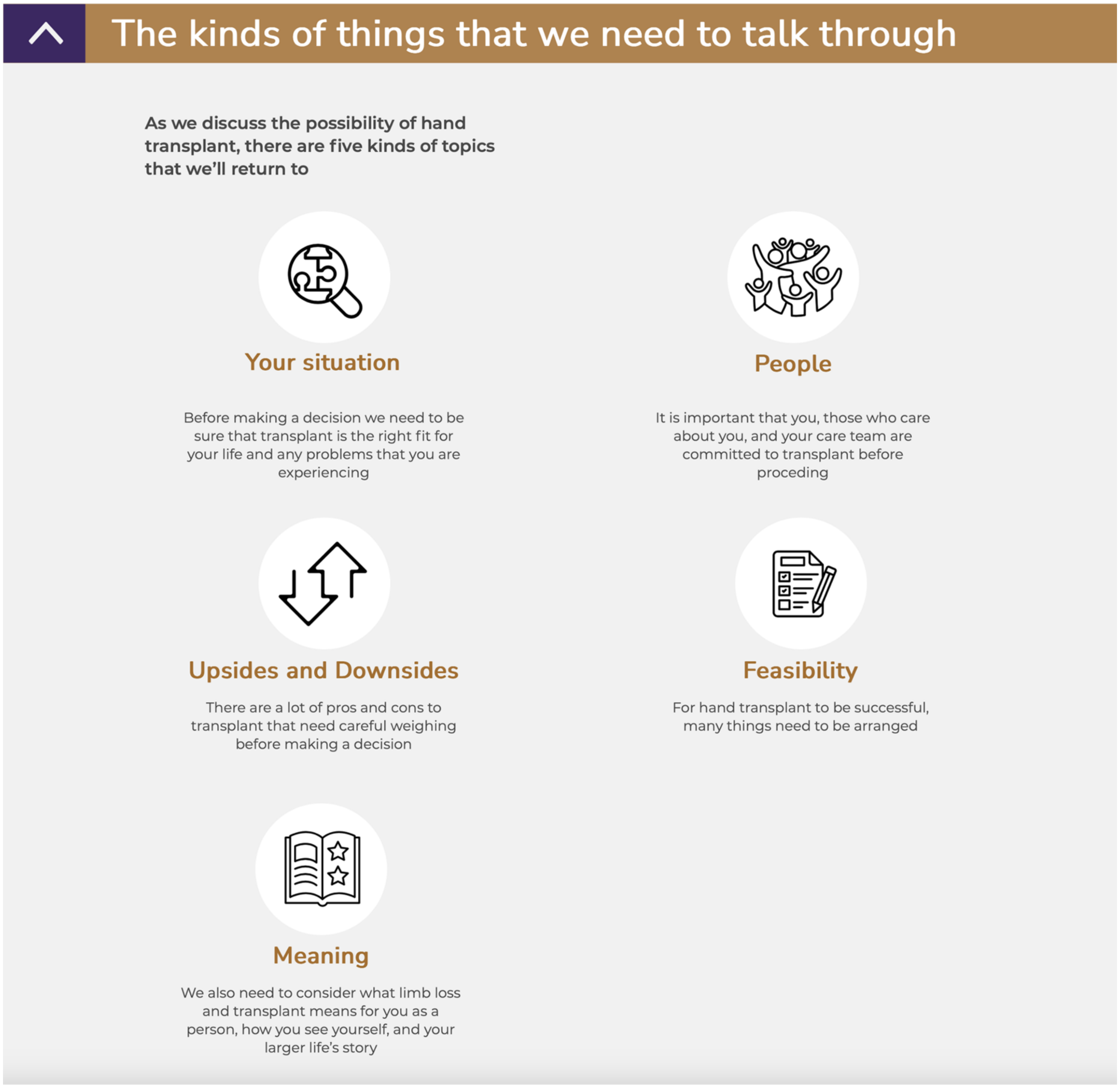

Supplementary Figure 3. The conversation aid presents information and points for reflection and conversation. The aid is organized around five topics that are aligned to Purposeful SDM issues.

- Your situation (issue: situations)
- People (issue: positions)
- Upsides and downsides (issue: effects)
- Feasibility (issue: situations)
- Meaning (issue: being)

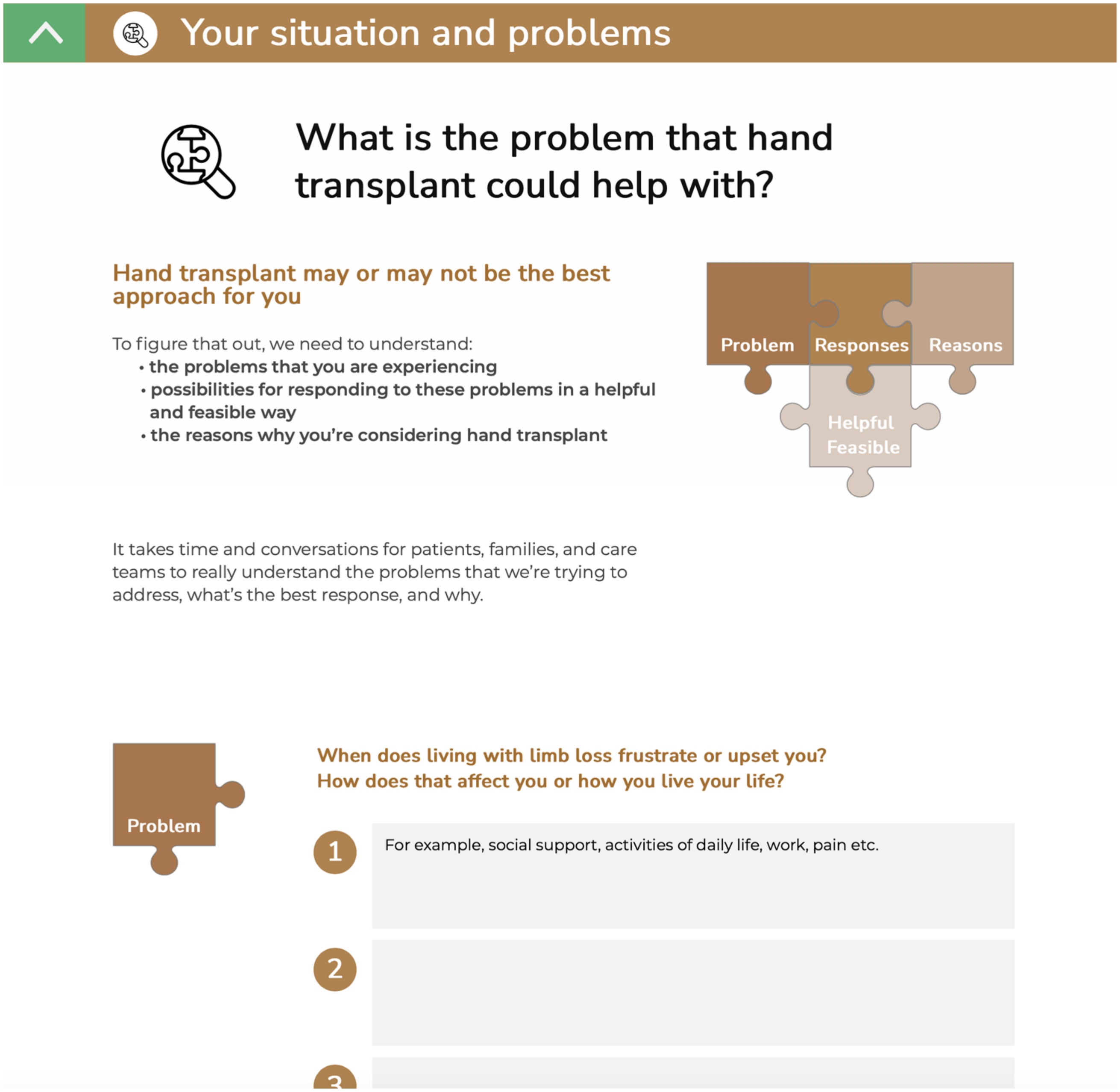

Supplementary Figure 4. Prototype conversation aid. (issue: situation)

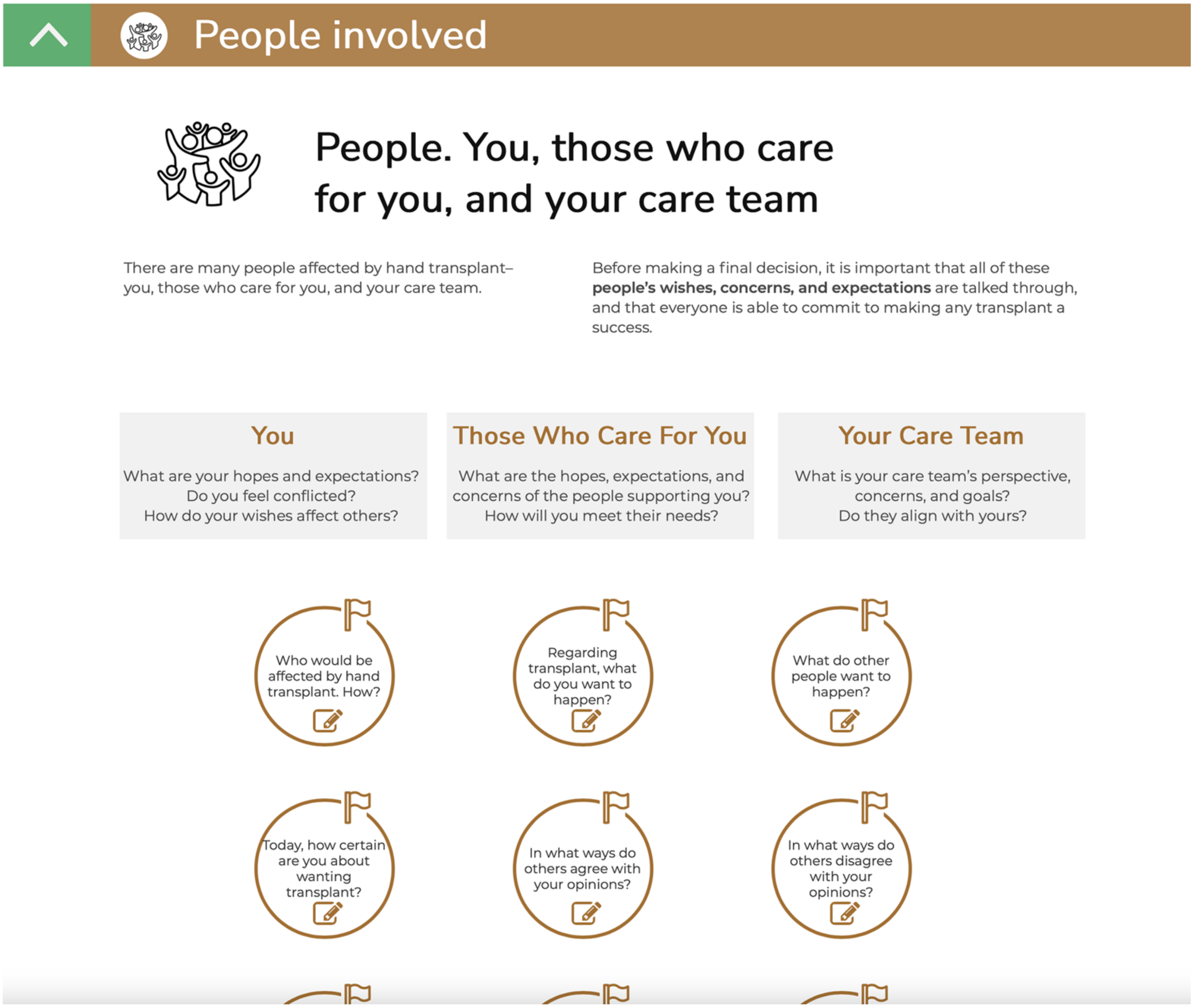

Supplementary Figure 5. Prototype conversation aid. (issue: positions)

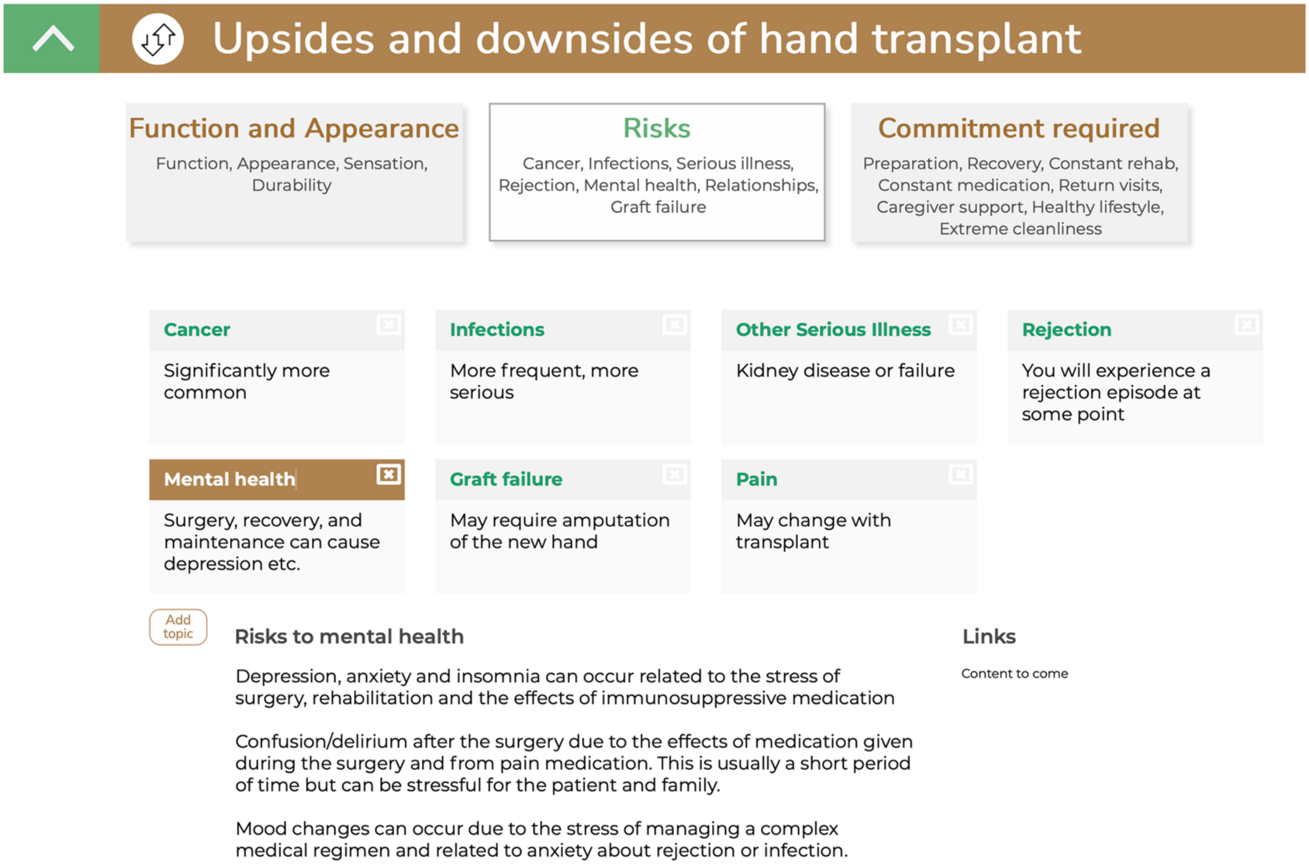

Supplementary Figure 6. Prototype conversation aid. (issue: effects)

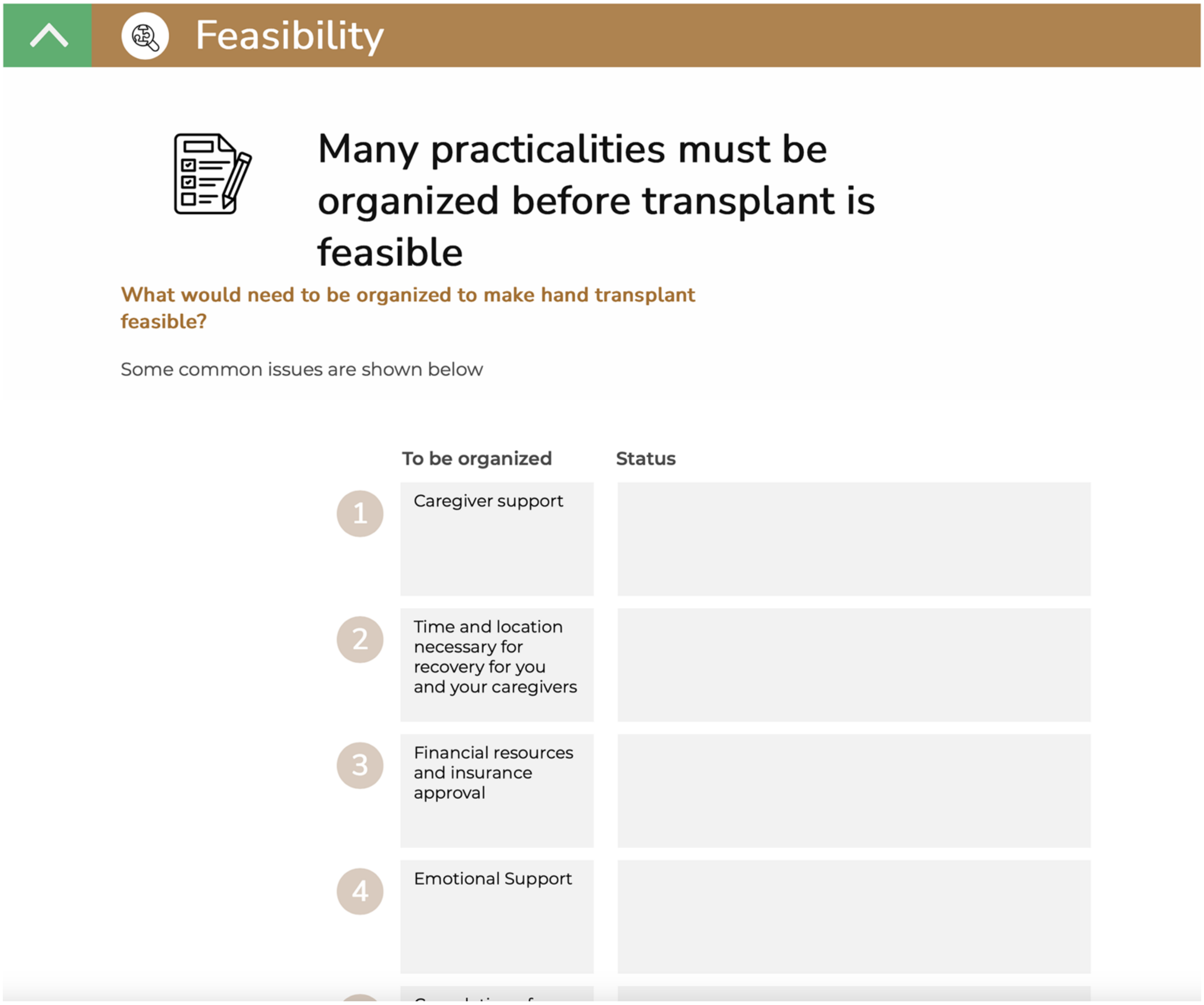

Supplementary Figure 7. Prototype conversation aid. (issue: situations)

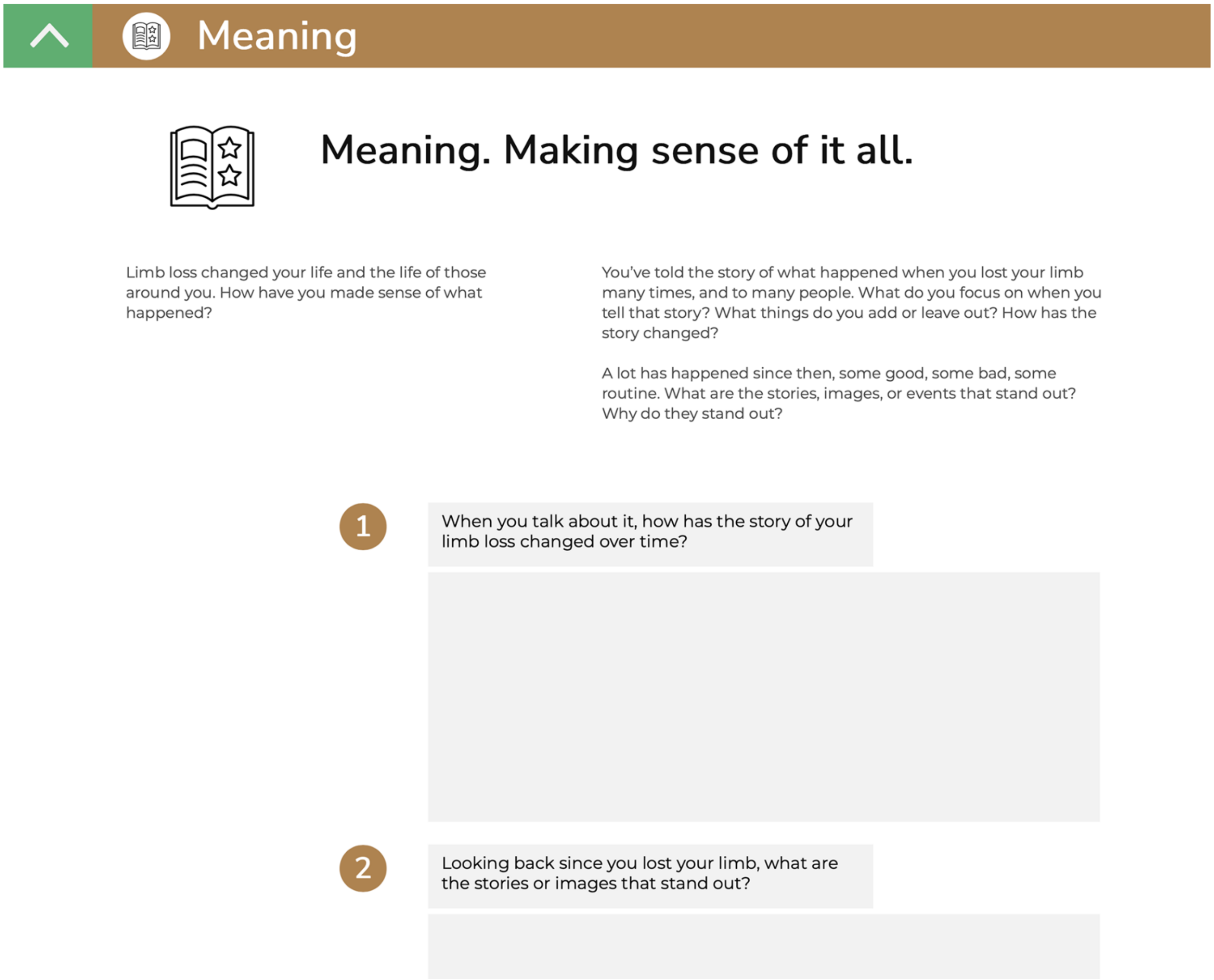

Supplementary Figure 8. Prototype conversation aid. (issue: being)
